# Antibody-related movement disorders – a comprehensive review of phenotype-autoantibody correlations and a guide to testing

**DOI:** 10.1186/s42466-020-0053-x

**Published:** 2020-02-20

**Authors:** Felix Gövert, Frank Leypoldt, Ralf Junker, Klaus-Peter Wandinger, Günther Deuschl, Kailash P. Bhatia, Bettina Balint

**Affiliations:** 1 Department of Neurology, Christian-Albrecht University of Kiel and University Medical Center Schleswig-Holstein, Kiel, Germany; 2 Neuroimmunology, Institute of Clinical Chemistry, Christian-Albrecht University of Kiel and University Medical Center Schleswig-Holstein, Kiel/Luebeck, Germany; 3 Department of Clinical and Movement Neurosciences, UCL Queen Square Institute of Neurology, London, UK; 4 Department of Neurology, University Hospital Heidelberg, Heidelberg, Germany

**Keywords:** Movement disorders, Antineuronal antibodies

## Abstract

**Background:**

Over the past decade increasing scientific progress in the field of autoantibody–mediated neurological diseases was achieved. Movement disorders are a frequent and often prominent feature in such diseases which are potentially treatable.

**Main body:**

Antibody-mediated movement disorders encompass a large clinical spectrum of diverse neurologic disorders occurring either in isolation or accompanying more complex autoimmune encephalopathic diseases. Since autoimmune movement disorders can easily be misdiagnosed as neurodegenerative or metabolic conditions, appropriate immunotherapy can be delayed or even missed. Recognition of typical clinical patterns is important to reach the correct diagnosis.

**Conclusion:**

There is a growing number of newly discovered antibodies which can cause movement disorders. Several antibodies can cause distinctive phenotypes of movement disorders which are important to be aware of. Early diagnosis is important because immunotherapy can result in major improvement.

In this review article we summarize the current knowledge of autoimmune movement disorders from a point of view focused on clinical syndromes. We discuss associated clinical phenomenology and antineuronal antibodies together with alternative etiologies with the aim of providing a diagnostic framework for clinicians considering underlying autoimmunity in patients with movement disorders.

## Background

Movement disorders are a common manifestation of a wide variety of diseases and aetiologies, many of which are neurodegenerative or genetic without truly disease-modifying treatment option. Autoimmune syndromes may mimic neurodegenerative or metabolic disorders, but are a treatable cause. Hence, their timely identification is of paramount importance.

Recent years have seen a rapid discovery of an increasing number of autoantibodies targeting neuronal (especially neuronal-surface) antigens. Within the antibody-specific subgroups, these diseases often display a fairly homogeneous phenotypic spectrum. Some movement disorders are so specific that their occurrence is a strong clue to the underlying autoantibody. Some syndromes are less specific but should raise the suspicion of a possible underlying autoimmune condition. Importantly, in all situations, a detailed history and careful examination often reveal clinical red flags, which help to guide diagnosis, initiate early causative immune-treatments and ultimately improve patient outcome.

The main focus of this review article is to guide syndrome-oriented approaches in patients with movement disorders, highlight clinical red flags and summarize the phenotypic spectrum of movement disorders of specific neuronal autoantibodies. Additionally, we critically discuss the specificity and sensitivity of antibody testing and whether detected antibodies are incidental or pathogenic.

## The clinical approach

A phenotype-orientated approach in movement-disorders first necessitates (1) the recording of clinical characteristics, (2) its phenomenological categorization and (3) a syndromic diagnosis [1]. Secondly, the movement disorder syndrome needs to be categorized into “isolated” or “combined” with other clinical symptoms [1]. In particular, the latter is important because neuronal antibody-associated diseases rarely occur with isolated movement disorders. Thus, accompanying clinical signs might be the necessary clue to reach the correct diagnosis. Below, we summarize in a syndrome-by-syndrome approach the relevant differential diagnoses and highlight red flags suggestive of an underlying autoimmune etiology.

### Cerebellar ataxia

Cerebellar ataxia is characterized by an impairment of coordination. Sporadic, progressive ataxia can be due to toxic (e.g. alcohol abuse), genetic (e.g. mitochondrial, trinucleotide expansion), metabolic (e.g. Niemann-Pick Type C) and autoimmune conditions. The latter usually present subacute over days to weeks and only rarely progress insidiously over many months. Similar to other categories of movement disorders, older age and the presence of multi-system symptoms should raise the suspicion of a paraneoplastic origin. Paraneoplastic cerebellar degeneration (PCD) usually presents with a subacute onset of rapidly progressive ataxia often combined with peripheral neuropathy, dementia, hearing loss and dysphagia [2]. Most patients exhibit severe disability early in the course of the disease, generally within 12 weeks [3]. In PCD, anti-neuronal antibodies are detectable in up to 60%. Most of these antibodies are directed against intracellular antigens (onconeuronal antibodies), although less commonly antibodies targeting cell surface antigens can be found as well. Almost all of the classical onconeuronal antibodies were described in the context of PCD [1], but the most common ones are anti-Yo (38%), anti-Hu (32%), anti-Tr (Delta/Notch-like-epidermal-growth-factor-related-receptor, DNER, 16%), and anti-Ri (12%) [4]. A relatively isolated cerebellar syndrome occurs in woman associated with Yo-antibodies and ovarian or breast cancer, or in males with Hodgkin lymphoma and Tr-(DNER)-antibodies [5, 6]. Otherwise, further clinical signs are frequently present and might help to guide diagnosis. For instance a more complex clinical syndrome with encephalomyelitis, limbic encephalitis, and peripheral sensory neuropathy is associated with small-cell-lung cancer (SCLC) and Hu-antibodies [7]. Opsoclonus might be seen in patients with Ri-antibodies and ovarian or breast cancer. The combination of proximal muscle weakness due to Lambert-Eaton mysathenic syndrome (LEMS) and ataxia may develop in patients with small-cell lung cancer (SCLC) and voltage-gated-calcium-channels (VGCC, mostly P/Q type) antibodies. A relatively common and usually idiopathic (non-paraneoplastic) autoimmune ataxia is associated with antibodies against the glutamate decarboxylase isotype 65 (GAD65), often associated with other autoimmune diseases like type 1 diabetes and thyroid disease [8]. Anti-GAD65 ataxia typically affects woman around 60 years but has been observed in young women and children. It usually presents with a slowly progressive or subacute course. A quarter of patients develop episodes of brainstem and cerebellar dysfunction or persistent vertigo several months before the onset of permanent cerebellar ataxia and therefore enter the differential diagnosis of episodic ataxia type 2 [8]. Clinically, most patients present with truncal ataxia, dysarthria, and nystagmus. Combined syndromes include additional GAD65-antibody-related neurological syndromes such as stiff-person-syndrome, limbic encephalitis and temporal-lobe epilepsy [8, 9].

Cerebellar ataxia is also one of the core symptoms of Contactin-associated-protein-2 (CASPR2)-antibody associated encephalitis and is seen in up to a third of patients [10, 11]. Of note, some patients might present with acute cerebellar ataxia without any other clinical symptoms [12, 13]. However, further clinical red flags like cognitive deficits, seizures, neuropathic pain, autonomic dysfunction and insomnia are present during the course of the disease. A striking feature in some patients are paroxysmal episodes of cerebellar ataxia resembling genetic episodic ataxia [14], albeit age of onset of CASPR2-antibody-syndromes is usually later in life. Isolated acute cerebellar ataxia was reported in association with antibodies targeting metabotropic glutamate receptor 1 (mGluR1) [15]. In nearly half of the reported patients dysgeusia was present, which might be a diagnostic clue [15]. Anti-Tissue-Transglutaminase-6 testing can guide diagnostics towards gluten-sensitive enteropathy, yet testing is only sensitive in patients who have not switched to gluten-free alimentation. In subacute and acute ataxic syndromes with brain stem involvement and prominent oculomotor dysfunction, anti-GQ1b testing for Miller-Fisher-spectrums disease is important.

Many more single cases and small series have been published with other neuronal autoantibodies, for some of which testing is only available in specialized research laboratories: anti-CV2/CRMP5, anti-ZIC4, anti-glycine receptor, anti-Ma proteins, Anti-Homer3, Anti-Carbonic anhydrase-related protein 8, TRIM9/67, septin-5, Anti-Sj/ITPR1, anti-PKCgamma, anti-GluRdelta2, anti-Ca/ARHGAP26, anti-Nb/AP3B2/beta-NAP [16]. Frequency of tumor association and clinical specificity is unclear for many of them. Most patients presented with acute ataxia, even though chronic progression was also seen [16]. As discussed below for other autoimmune movement disorders, in clinically suspected autoimmune ataxia with negative testing for frequent antibodies, a tissue-based screening test for “anti-cerebellar” immunoreactivity in a specialized research laboratory is considered a more promising second diagnostic step than testing these rare antibodies individually.

In summary, the clinical presentation of autoimmune ataxia can be broad but generally a subacute onset in combination with further clinical symptoms suggestive of limbic encephalitis, preceding episodes of transient brainstem and cerebellar dysfunction, fluctuating muscle stiffness and spasms, dysautonomia, insomnia, dysgeusia and infranuclear opthalmoplegia indicate a potential autoimmune pathology. A paraneoplastic origin should be considered in patients with a rapid progressive cerebellar syndrome with coexisting subacute sensory neuropathy, cognitive decline, hearing loss, fluctuating proximal muscle weakness or opsoclonus.

### Chorea and dyskinesias

Chorea is a hyperkinetic movement disorder characterized by brief irregular purposeless movements that flit and flow from one body part to another. It can occur isolated or associated with athetosis (choreoathetosis) and other hyperkinetic movement disorders which are often summarized under the umbrella term dyskinesias. Accordingly, both conditions are grouped together below. Prominent chorea can be caused by a large variety of diseases which can be broadly divided into inherited and acquired conditions. While Huntington’s disease is by far the most common cause for adult-onset chorea, autoimmune diseases are actually the second most common acquired cause for chorea after vascular lesions [17]. Prime examples for the latter are Sydenham’s chorea and chorea in antiphospholipid syndrome or systemic lupus erythematosus [1]. However, in recent years several antineuronal antibodies were described as a cause of autoimmune chorea and dyskinesias.

Distinct and complex hyperkinetic movements affecting the mouth and the limbs are a characteristic clinical feature of anti-N-Methyl-D-Aspartate-receptor (NMDAR) encephalitis which is by itself the most frequent antibody-associated autoimmune encephalitis [18, 19]. Complexity mostly relies on the coexistence of chorea, dystonia and stereotypies which are seen in up to 90% of patients with anti-NMDAR encephalitis and might present as an early clinical feature or even presenting symptom [19]. However, the clinical presentation differs between children and adults. While children typically present with movement disorders and seizures, adults present mainly with behavioral changes, psychiatric disorders or cognitive impairment [20]. A recent study demonstrated that young children frequently present as the first clinical sign of NMDAR-ab encephalitis with transient unilateral dystonic or tonic posturing of the hand or the foot as the first evidence of focal epilepsy [20]. Eventually, generalized chorea or stereotypical movements together with mutism and orofacial dyskinesias might develop. Children might also present with prominent generalized chorea or hemichorea and behavioral changes, leading to the misdiagnosis of Sydenham’s chorea, even though additional neurological signs like new-onset seizures, ataxia or cognitive deficits are typically present and should caution against such a misdiagnosis [21, 22]. Furthermore, children may develop NMDAR-antibody encephalitis after herpes simplex virus encephalitis (HSVE; the viral encephalitis probably being a trigger of brain autoimmunity) and may manifest with chorea or ballism [23]. However, monosymptomatic manifestation of anti-NMDAR encephalitis is extremely rare and movement disorders are typically combined with other signs (e.g. cognitive dysfunction, seizures, prominent psychiatric disturbance, dysautonomia) that are red flags for the right diagnosis. Of note, a similar clinical picture with prodromal fever, headache, or gastrointestinal symptoms, followed by confusion, seizures, decreased level of consciousness and orofacial dyskinesias was described in (mostly female) patients with neurexin-3a-antibodies [24]. However, encephalitis with neurexin-3a antibodies seems to be rare.

Similarly, antibodies against the striatal dopamine receptor 2 (D2R antibodies) appear to be a rare cause of pediatric movement disorders in children. So far, D2R-antibodies were detected in children with basal ganglia encephalitis, Sydenham’s chorea or in choreoathetoid relapses after HSVE [25, 26]. These findings await, however, confirmation from other laboratories, as well as determination of their specificity.

Chorea or hemichorea was also described in the context of leucine-rich-glioma-inactivated-1 (LGI1)- and CASPR2-encephalitis, sometimes as the main clinical or presenting symptom [17, 27, 28].

Finally, IgLON5-antibodies are associated with a variety of neurological symptoms, including chorea or orofacial dyskinesias [29, 30]. Clinical red flags which should alert the clinician to test for IgLON5 antibodies are prominent sleep disorders, in particular a non-rapid eye movement (NREM) sleep parasomnia with simple and finalistic movements, breathing difficulties (related or unrelated to sleep; stridor), cognitive decline, and bulbar symptoms [30].

Autoimmune, mostly paraneoplastic chorea can also be associated with antineuronal autoantibodies targeting intracellular, intranuclear antigens. This is more likely in older male patients with generalized chorea and other coexisting clinical signs including weight loss and peripheral neuropathy [17]. The most common antibody associated with paraneoplastic chorea is Collapsin-response-mediated protein-5 (CRMP-5) usually related to SCLC or thymoma [31–33]. Typically, chorea associated with CRMP-5 antibodies is part of a multifocal clinical syndrome including encephalopathy, ataxia, optic neuritis, peripheral neuropathy and, rarely, myelopathy [31, 33]. Imaging might demonstrate FLAIR hyperintensities in the basal ganglia, limbic regions, brainstem, and white matter [32]. Another antibody described in the context of paraneoplastic chorea is anti-Hu (also known as ANNA-1), which is also frequently associated with SCLC.

In summary, subacute chorea with prominent orofacial involvement combined with stereotypies, dystonia, neuropsychiatric symptoms, seizures and other signs of encephalopathy, particularly in children and young adults, favor the diagnosis of anti-NMDAR encephalitis. Further clinical red flags for an autoimmune or paraneoplastic chorea are subacute cognitive decline, progressive neuropathy and ataxia, weight loss, behavioural changes, dysautonomia, prominent sleep behaviour disorders and bulbar symptoms.

### Dystonia

Dystonia is defined as a movement disorder characterized by sustained or intermittent muscle contractions causing abnormal, often repetitive, movements, postures, or both. Dystonia might be the only clinical finding (isolated dystonia) or can be combined with other movement disorders (combined dystonia) or the occurrence of other neurological signs. Dystonia can be caused by numerous different causes and can generally be divided into acquired (e.g. brain lesion), inherited (e.g. due to *TOR1A* mutations, DYT-TOR1A/DYT1) or idiopathic (e.g. idiopathic cervical dystonia) causes. Antibody-associated dystonia typically presents as a combined dystonia, i.e. in combination with further clinical signs, in particular encephalopathy. A presentation of autoimmune encephalitis as isolated dystonia is extremely unusual. In children and young adults, dystonia manifesting as hemidystonia or craniocervical dystonia were reported as prominent clinical signs in association with NMDAR-antibodies [34, 35]. Oromandibular and limb localization of dystonia is most common, although oculogyric crises are possible and rarely even generalized dystonia has been observed [36]. In general, movement disorders including dystonia are more common in children than in adults with NMDAR encephalitis and the presence of dystonia in a patient with subacute onset of encephalopathy should prompt consideration of NMDAR-antibody testing [37].

Dystonia is less common in other autoimmune syndromes. In adults, severe jaw-closing dystonia often combined with recurrent episodes of laryngospasms has been described in 20% of patients with Ri-antibodies and seems to be almost pathognomic for Ri-associated paraneoplastic brainstem encephalitis [38, 39]. This condition often results in severely impaired nutrition, respiratory distress and even death. Imaging can be normal or demonstrate T2 hyperintensity in the pons and temporal regions. Most patients are females with breast cancer [38]. Jaw and cervical dystonia might rarely be seen in patients with IgLON5-antibodies, but with further classical signs of IgLON5-disease like gait instability, bulbar symptoms and sleep abnormalities [29].

In summary, red flags for an autoimmune cause of dystonia are subacute onset and additional signs. In children and young adults, particularly if combined with further movement such as orofacial dyskinesia, chorea and stereotypies, and additional signs like encephalopathy and psychiatric features, NMDAR-antibody testing is mandatory. In adults, the combination of jaw dystonia and breathing difficulties should prompt testing for Ri-antibodies. In other autoimmune syndromes, dystonia is not a prominent or distinguishing feature and other signs are more likely to guide the diagnosis.

### Myoclonus

Myoclonus is defined by sudden, brief and involuntary jerks. Beside toxic, metabolic and infectious causes, several autoantibody-associated neurological diseases might present with a subacute myoclonus. However, further characteristic clinical signs are usually present - for instance, characteristic eye movement abnormalities in opsoclonus-myoclonus syndrome (OMS); hyperekplexia and prominent brainstem and autonomic involvement in progressive encephalomyelitis with rigidity and myoclonus (PERM); and clinical signs for limbic encephalitis (seizures, memory impairment) in LGI1- and CASPR2-encephalitis [40]. Isolated myoclonus is only rarely indicative of underlying autoimmunity but should be considered when common causes like toxic, neurodegenerative, structural and genetic conditions are excluded.

Myoclonus is one of the defining clinical characteristics in patients with opsoclonus-myoclonus syndrome (OMS). Opsoclonus is clinically characterized by rapid, involuntary, multivectorial conjugate fast eye movements without intersaccadic intervals. Sometimes opsoclonus can be mild, suppressed by fixation upon examination and only be observable for a short time immediately after reopening of the eyes. Myoclonus predominantly affects the limbs but can also present as axial myoclonus. OMS is often accompanied by ataxia, behavioral changes or sleep disturbances [41]. In children, OMS is commonly associated with neuroblastoma, while in adults it may be of (in order of frequency) postinfectious, idiopathic or paraneoplastic origin [42]. Paraneoplastic etiology should be suspected particularly in the elder and if encephalopathy is an accompanying feature. Younger patients (below 40) only rarely have underlying tumors, but there is an entity of ovarian teratoma associated OMS which should not be overlooked [43]. In most cases of OMS, no underlying autoantibodies can be detected. Occasionally, Ri-antibodies can be found and are highly suggestive of ovarian or breast cancer. Recently, OMS in the context of NMDAR, GABA(b), GlyR-antibodies, and more recently, yet not reproduced by other centers, glutamate receptor δ2 antibodies, have been described [44, 45]. Importantly, these antibodies are not OMS-specific [1].

Distinct forms of myoclonus occur with CASPR2-encephalitis [46, 47]. For example, prominent myoclonus of the lower limbs while standing or walking in elderly men is an emerging phenotype associated with these antibodies [48, 49]. Similarly, segmental spinal myoclonus leading to trunk flexion or abdominal wall contractions different from (mostly functional) propriospinal myoclonus can be a distinct presentation of patients with CASPR2 antibodies (authors’ observation). Nevertheless, most patients manifest with classic limbic encephalitis, Morvan syndrome or neuromyotonia. Patients with LGI1-antibodies rarely have actual myoclonus but so-called faciobrachial-dystonic seizures (see below, Paroxysmal movement disorders) [50] which were probably frequently mistaken as myoclonus in several earlier reports [46, 47].

Myoclonus, typically together with hyperekplexia, are key clinical components in patients with progressive encephalomyelitis with rigidity and myoclonus due to glycin receptor (GlyR) antibodies (PERM, see Stiff-person spectrum disorders below). Similarly, myoclonus with or without hyperekplexia, can be a prominent clinical finding in Dipeptidyl-peptidase-like protein-6 (DPPX) antibody-associated disease. The clinical spectrum of associated features is broad, but prodromal, prolonged and severe diarrhea leading to considerable weight loss [51, 52] is a characteristic red flag pointing to the diagnosis.

Other than the above, myoclonus is rarely a prominent sign of neuronal autoimmunity. Very rarely, it has been reported in patients with IgLON5 autoantibodies, usually in combination with a distinct sleep disorder, bulbar dysfunction and gait abnormalities [29]. Recently, antibodies against glial-fibrillary-acidic-protein (GFAP) have described to define an autoimmune astrocytopathy manifesting with meningoencephalomyelitis with headache and subacute encephalopathy [53, 54]. Later reports expanded the clinical spectrum reporting prominent myoclonus and/or tremor in the course of the disease [55, 56]. Concomitant autoimmunity (also paraneoplastic) is frequent with GFAP-antibodies, and further studies will be required to conclusively investigate specificity of these antibodies and the associated clinical spectrum.

In summary, subacute onset of myoclonus in combination with signs of encephalopathy, seizures, brainstem involvement, autonomic features and recent prominent sleep disorder should alert the clinician for an autoimmune condition, and testing in particular for CASPR2, LGI1, DPPX, IgLON5 and GlyR antibodies should be considered. Elderly man with prominent myoclonus of the lower limbs or segmental spinal myoclonus, even in the absence of further obvious clinical signs, should be tested for CASPR2 antibodies.

### Parkinsonism

Parkinsonism is defined as bradykinesia in combination with either rest tremor, rigidity, or both, and the by far most common cause is idiopathic Parkinson’s disease (PD). PD is further characterized by a clear beneficial response to levodopa, a classic rest tremor and an asymmetric appearance of motor symptoms. In contrast, “atypical parkinsonism” is suspected when clinical signs not compatible with the diagnosis of idiopathic PD are present and response to levodopa is poor. The most common causes are other neurodegenerative diseases like progressive supranuclear palsy (PSP), corticobasal degeneration (CBD), or multisystem atrophy (MSA), and less frequently infectious, toxic or metabolic causes. Autoimmune parkinsonism is rare and generally presents as an atypical parkinsonian syndrome, usually reflecting brainstem encephalitis with eye movement abnormalities or other brainstem signs, and sleep disorders [57].

Paraneoplastic parkinsonism has been reported with CRMP5- and Ma2- (rarely Ri-) antibodies [39, 58–60]. Most cases present with a rapid progressive and disabling course. Anti-Ma2 encephalitis typically presents with progressive limbic, diencephalic or brainstem dysfunction [60]. Classic red flags are hypothalamic-pituitary dysfunction, weight gain, prominent sleep disorders including excessive daytime sleepiness, rapid eye movement (REM) sleep behaviour disorder (RBD), narcolepsy-cataplexy, and eye movement abnormalities, in particular vertical gaze palsy [60]. The latter might mimic PSP, especially when patients display apraxia of lid opening, yet the disease progression and additional signs would caution against such a misdiagnosis [60]. Imaging typically demonstrates thalamic and hypothalamic T2 hyperintensities in anti-Ma2 encephalitis, while involvement of the basal ganglia is more characteristic for CRMP5-antibody encephalitis [60].

Nonparaneoplastic autoimmune parkinsonism was described in the context of several different antibodies. For instance, patients with IgLON5 antibodies can present with a clinical picture of postural instability and gaze palsies, resembling PSP in up to 23% [30]. However, concurrent sleep abnormalities like parasomnia, sleep apnea, insomnia, or excessive daytime sleepiness are red flags for IgLON5-related disease in nearly all patients with IgLON5 encephalitis.

Further autoimmune parkinsonism mimicking PD, MSA or PSP was reported in patients with LGI1-, CASPR2-, and DPPX-antibodies [52, 61–63]. Patients with stiff-person-spectrum-disorders (SPSD, see below) with glycine-receptor-alpha-1 (GlyRα1) or GAD antibodies may appear parkinsonian. In children with acquired parkinsonism testing for NMDAR antibodies and D2R antibodies should be considered [25, 35].

In summary, red flags for autoimmune or paraneoplastic parkinsonism include a subacute onset, clinical signs of a coexisting encephalopathy and symptoms suggestive of prominent brainstem involvement like abnormal eye movements, severe sleep abnormalities, early and prominent dysarthria, dysphagia and postural instability.

### Paroxysmal movement disorders

Paroxysmal movement disorders are a rare group of predominantly genetically determined inherited disorders characterized by self-limiting episodes of abnormal movements. Both paroxysmal dyskinesias and episodic ataxias have in common that onset is in child- or early adulthood and mostly have an autosomal-dominant inheritance. In contrast, the so far reported antibody-associated paroxysmal movement disorders usually have an onset later in life.

By far the best characterized antibody-associated paroxysmal dyskinesias are faciobrachial dystonic seizures (FBDS) [64, 65]. FBDS are characterized by a distinctive clinical phenotype with brief (typically < 3 s) but extremely frequent (up to hundreds per day) episodes of stereotypical dystonic posturing of the unilateral face, arm, and occasionally leg or combinations. In some cases, FBDS last longer and simultaneous bilateral involvement can result in drop attacks [64, 65]. The recognition of this characteristic clinical phenotype is important because FBDS are highly specific for LGI1-encephalitis and typically occur early and often before onset of limbic encephalitis. According to a recent publication, MRI demonstrates basal ganglia hyperintensities on T1- or T2-weighted sequences in about 40% of patients [66], although in the authors’ experience, such MRI abnormalities are less prevalent. Prompt immunotherapy should be initiated because it may prevent development of the full-blown encephalopathic picture with cognitive impairment. Of note, antiepileptic treatment without immunotherapy is mostly ineffective [64].

Paroxysmal brief dystonic arm posturing and paroxysmal exercise-induced foot weakness was described in single patients with anti-NMDAR encephalitis [67, 68]. Painful tonic spasms (PTS) can mimic paroxysmal dyskinesias and are commonly seen in demyelinating diseases. Interestingly, PTS is more frequent in neuromyelitis optica spectrum disorders (NMOSD) with AQP4 antibodies than in multiple sclerosis and is seen in over 20% of patients with NMOSD [69]. Myelin-oligodendrocytic glycoprotein (MOG)-antibody related NMOSD is associated less frequently with tonic spasms [70].

A clinical picture resembling paroxysmal episodic ataxia type 1 can occasionally be seen in patients with autoimmune encephalitis with CASPR2 antibodies [14]. In most patients, orthostatism and walking provoked recurrent attacks. A further case of a patient with paroxysmal onset of myoclonus of the lower limbs with slight dystonic posturing while walking was described in a patient with CASPR2 antibodies [48].

In summary, sudden brief and repetitive dystonic posturing of the face and the limbs should trigger LGI1-antibody testing. In young women, painless paroxysmal dystonic posturing should prompt consideration of NMDAR-antibody testing whereas painful tonic spasms warrant testing for NMOSD-associated antibodies (AQP4, MOG). Elder man with paroxysmal episodes of ataxia and myoclonus in particular with clinical evidence of coexisting limbic encephalitis or neuromyotonia should be tested for CASPR2-antibodies.

### Stiff person spectrum disorders

Stiff-person-spectrum disorders (SPSD) are a group of rare diseases (estimated prevalence: 1/1.000.000), characterized by the clinical core features of stiffness, spasms and hyperekplexia. They also share electrophysiological characteristics (continuous motor unit activity, CMUA; enhanced exteroceptive reflexes), pathological findings, and a range of associated antibodies [71]. They differ in the distribution of stiffness and the presence or absence of other neurological signs:

The classic form, stiff-person syndrome is characterized by muscle stiffness and superimposed painful spasms involving trunk and proximal limb muscles, often with a characteristic lumbar hyperlordosis, and a stiff, wooden gait. In stiff-limb syndrome (SLS), in contrast, stiffness is more distal and confined to a limb (leg, arm). There are variants with more widespread involvement, such as stiff-person plus with additional neurological signs like cerebellar ataxia or epilepsy. In progressive encephalomyelitis with rigidity and myoclonus (PERM), stiffness may be generalized and there are additional brainstem signs such as oculomotor disturbance with gaze palsies, nystagmus and ptosis, and bulbar symptoms such as dysphagia, dysarthria and trismus and frequently, prominent autonomic symptoms or even autonomic failure, including respiratory failure [72].

Most SPSD patients harbor antibodies against GAD65 in the serum and cerebrospinal fluid. GAD65 antibodies associate also with cerebellar ataxia (and less frequently with focal epilepsy) and of course with diabetes type 1; thus, these are often comorbidities in GAD-antibody positive SPSD patients, just as autoimmune thyroid disease or vitiligo. GlyR-antibodies are the second most frequent antibody in SPSD and occur slightly more frequent in PERM, even though classic SPS with GlyR-antibodies exists [73]. GlyR-antibodies associate with thymomas, and thymoma removal is key in such cases. Amphiphysin-antibodies are less frequent in SPSD, but important as they strongly associate with breast and lung cancer. The clinical spectrum of anti-amphiphysin syndromes is broad and encompasses, besides SPSD, also cerebellar ataxia, limbic encephalitis, sensory ganglionopathy and myelopathy. DPPX-antibodies also have a broad clinical spectrum and can give rise to combined SPSD variants [74, 75]. Different to the classic lumbar hyperlordosis, anti-DPPX patients may develop stiffness more of the upper trunk with scoliosis. Additional features comprise cerebellar signs, gastrointestinal symptoms (in particular, long-lasting diarrhea), cognitive dysfunction and dysautonomia.

In summary, stiffness, spasms and acquired hyperekplexia call for antibody testing. Associated features and autoimmunity may indicate the underlying antibody (e.g. GAD-antibodies with coexisting type 1 diabetes), but there is much clinical overlap also between the various antibodies, and sometimes co-existence of two different antibodies.

### Tics

Tics are rapid, brief and stereotyped involuntary movements that are often preceded by a premonitory urge and can be voluntarily suppressed. They can present as simple motor or vocal tics like eye-blinking or sniffing or can be more complex designating sequences of stereotyped movements, or words or phrases. Tics are most often recognized in the context of primary tic disorders like Tourette’s syndrome but are rarely also encountered secondary to neurometabolic (e.g. Lesch-Nyhan syndrome) or neurodegenerative disorders (e.g. Huntington’s disease), drugs or toxins. The role of antibodies in tic disorders remains controversial. Tics are part of the rather complex and controversial clinical entity known as paediatric autoimmune neuropsychiatric disorders associated with streptococcal infections (PANDAS), where antibodies modulating dopamine D1 and D2 receptors have been hypothesized as being causative; however, this has not been independently confirmed [25, 76]. To date, no antineuronal antibody has been consistently shown to underlie tic disorders. Hence, the authors would caution against serum antibody testing in pure tic disorders and encourage critical interpretation of antibody findings and repeat testing in different laboratories in “seropositive” tic disorders.

### Tremor

Tremor is a rhythmic sinusoidal oscillation of a body part, usually due to alternate activation of agonist and antagonist muscles. The differential diagnosis of tremor is broad and includes neurodegenerative, genetic, metabolic, infectious, toxic and other causes. An isolated tremor is highly unlikely to be due to an antibody-mediated disease. However, tremor can occur as part of an autoimmune encephalitis, and has been described with various antibodies typically in the context of a widespread encephalopathy: AMPAR-, CASPR2-, LGI1-, DPPX-, GABAR-B-, GlyR-, mGluR1-, NMDAR-, and GFAP-antibodies [35, 47, 51–54]. GFAP-antibodies have been described associated with a distinctive, corticosteroid-responsive, autoimmune meningoencephalomyelitis and tremor, myoclonus and ataxia were frequently reported during the course of the disease [53, 54, 56]. Interestingly, a striking perivascular radial enhancement mimicking vasculitis was found on MRI in over half of the patients [54]. Specificity of this antibody has not been confirmed by independent laboratories yet.

Patients presenting with an isolated “whole-body tremulousness” are often mistaken as generalized tremor and may actually suffer from generalized repetitive myoclonus [40]. In such patients CRMP5- and LGI1−/CASPR2-antibodies were detected [40]. Beside the classic antineuronal antibodies causing autoimmune encephalitis several antibodies targeting proteins adjacent to the node of Ranvier (paranodal), such as contactin-1 (CNTN1), neurofascin-155 (NF155), nodal neurofascin (NF140/186) and contactin-associated-protein-1 (CASPR1) were described in patients who developed a disabling tremor in the context of CIDP [77, 78].

In summary, autoimmune tremor is seen in patients with various encephalitides, or in the context of paranodal-antibody-associated CIDP.

## Antibody testing

**Fig. 1:**
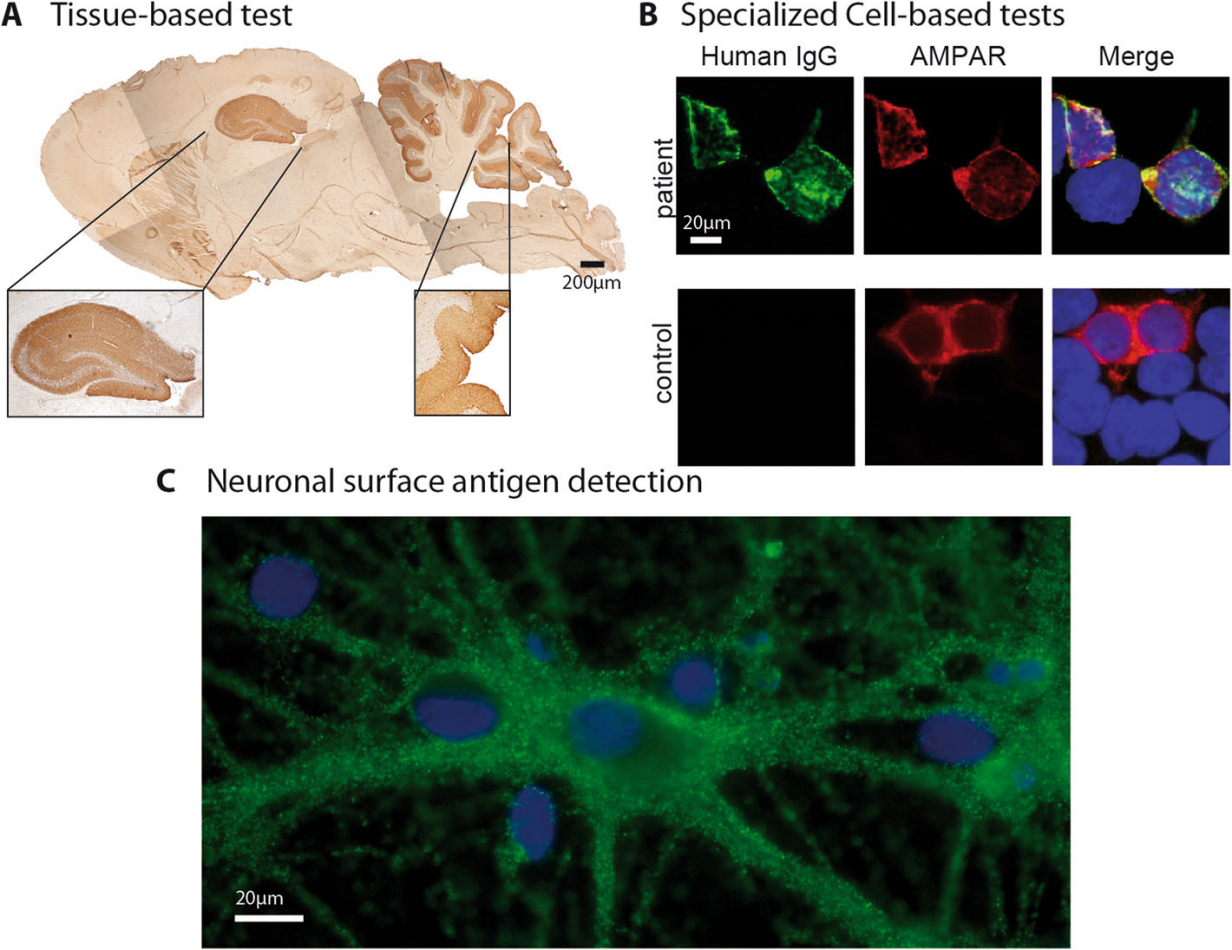
Example of comprehensive aurtoantibody testing available in scientiifc research settings. **a** Tissue-based test using sagittal rat brain sections optimized for detection of neuronal surface antibodies. Brown staining indicates specific binding of human IgG. Hippocampal and cerebellar regions are shown magnified. Shown is an example of the staining obtaining with GABA(A) receptor antibody containing patient CSF. **b** Cell-based assay with HEK293 cells expressing human autoantigens. This example shows cells transfected with AMPA receptor subunits stained with serum of a patient with anti-AMPA receptor encephalitis. Green staining indicates human IgG, red staining indicates a commercial antibody detecting transfected cells. Merged images are shown on the right side. Yellow indicates cells transfected and detected by human IgG thus indicating presence of autoantibodies targeting the transfected antigen. In addition to the shown example of cells fixed with paraformaldehyde, for some autoantibodies non-fixed live-cell based assays, which stain cells before addition of fixatives are more sensitive for the detection of some autoantibodies (e.g. MOG, not shown). **c** Primary, embryonal, rat hippocampal neurons are stained with serum from a patient with anti-AMPA receptor encephalitis. Cells are alive and non-permeabilized so that only surface antigens are detected by autoantibodies. Green indicates human IgG binding to individual synapses, blue is a DAPI counterstaining of nuclei to demonstrate presence of neurons

Comprehensive serological testing for autoantibodies can be done by a combination of (1) immunoblot, (2) Enzyme-linked-immune-tests (e.g. ELISA) and/or radioimmunoassay (RIA) (3) cell-based assays (CBA) and (4) tissue-based assays (TBA). These tests can be complemented by additional tests in research laboratories using live-cell CBAs and non-permeabilized (live) cells and primary hippocampal neurons (Fig. 1).

In general, testing should follow five rules: (a) Always use a combination of antigen-specific tests (immunoblots, ELISA/RIA, CBAs) and tissue-based screening tests (TBA). (b) Include immunoblots for intracellular antigens, CBAs for extracellular antigens, and ELISA/RIA for GAD65, VGCC. (c) The combination of results obtained from testing serum and CSF improves sensitivity and specificity for the majority of antibodies and prevents false-positives and false-negatives (not uncommon if testing serum only). (d) Testing should start with the common antibodies. If these remain negative and clinical suspicion of autoimmune etiology remains high, in a second diagnostic step specialized TBAs and CBAs in research laboratories can be utilized to screen for less common and unknown antibodies. (e) Nevertheless, seronegativity in spite of comprehensive testing is common, especially in sporadic ataxia and atypical syndromes with a low a priori probability of autoimmune etiology. A summary of common and uncommon antibodies in movement disorders stratified by syndrome is given in Table 1. In everyday practice, the significance of incidental antibody findings in patients with movement disorders can be difficult to judge. Importantly, serum testing using a commonly employed battery of CBAs yields up to 1% of false positive results at low titers using serum (authors’ observation). Clues to unspecific findings not-related to the patients’ conditions are: Very low titers in serum, e.g. CASPR2 antibodies at 1:10 in serum; atypical clinical presentation, e.g. typical Parkinson syndrome without atypical features and anti-NMDAR antibodies at low titers in serum; antibody not found in CSF, e.g. low titer CASPR2 antibody in serum 1:10 but not in CSF. However, this needs to be interpreted with caution, since some antibodies like LgI1 often test negative in CSF using standard CBAs. Interpretation of GAD65 testing is especially challenging. Testing can be done using different systems (ELISA, RIA and TBA) and results are not directly comparable. In general, mostly very high titers in serum (e.g. > 2000 IU/ml in ELISA), positive GAD65 abs in CSF and positive GAD65-specific results on TBAs are highly associated with neuronal syndromes and indicate potentially treatment-responsive patients. Finally, serum antibody results which remain unclear should be considered to be retested in a scientific laboratory using specialized test systems for confirmation, which often improves specificity.

**Table 1:** Antibody-related movement disorders: Clinical features and tumor association

Syndrome	Antigenic targets of associated antibodies	Specific movement disorder features	Other possible clinical features	Tumor association
Ataxia	GAD	Mostly truncal ataxia, nystagmus and dysarthria typically in woman over 60; preceding episodes of brainstem and cerebellar dysfunction or persistent vertigo before onset of permanent ataxia in some patients	Often associated with further autoimmune diseases e.g. diabetes type 1, thyroiditis. Overlap with stiff-person syndrome, limbic encephalitis temporal-lobe epilepsy	< 5%
	CASPR2	Rarely isolated ataxia, generally combined ataxia with→ Rarely presentation of paroxysmal episodic ataxia (generally in the setting of limbic encephalitis)	Ataxia, pain, sleep dysfunction, autonomic dysfunction, weight loss, limbic encephalitis; male predominance (85%); age > 65 yrs.	∼20% (mostly thymoma)
	DPPX	Combined ataxia with→	Dysautonomia, pyramidal signs, sensory symptoms, cognitive problems. Red flags: Prolonged diarrhea, weight loss	< 10%, lymphoma
	NMDAR	Combined ataxia with→ Ataxia is more frequent in children	Behavioral changes, psychiatric disorders, cognitive impairment, seizures, mutism, dysautonomia	25–50% of woman have ovarian teratomas. In children very rare. In patients > 45 yrs. Other tumors possible, e.g. SCLC, breast cancer, etc
	IgLON5	Combined ataxia with→	Sleep disorder, bulbar dysfunction, gait abnormalities, cognitive decline, eye movement abnormalities	< 5%
	mGluR1	Isolated acute cerebellar ataxia	In 50% of patients dysgeusia	Unknown, possibly ∼50% lymphoma
	VGCC	Isolated paraneoplastic cerebellar ataxia or combined with→	Lambert-Eaton syndrome or limbic encephalitis	Highly associated with SCLC especially if associated with SOX1 abs
	GQ1b	Combined ataxia with→	areflexia, ophthalmoplegia and further signs for brainstem involvement (Miller-Fisher Syndrome/ Bickerstaff encephalitis)	< 5%
	Yo/CDR2	Isolated or combined paraneoplastic cerebellar ataxia with→	brainstem encephalitis, neuropathy	> 95%, highly associated with breast and ovarian cancer
	Hu/ANNA1	Combined paraneoplastic cerebellar ataxia with→	encephalomyelitis, limbic encephalitis, peripheral sensory neuropathy	> 95%, highly associated with SCLC and other neuroendocrine tumors.
	Ri/ANNA2	Combined paraneoplastic cerebellar ataxia with→	limbic or brainstem encephalitis, myelitis and opsoclonus	95%, highly associated with breast and ovarian cancer
	Tr/DNER	Isolated paraneoplastic cerebellar ataxia or combined with→	encephalopathy or neuropathy	95%, highly associated with lymphoma
	PCA2	Combined paraneoplastic cerebellar ataxia with→	limbic or brainstem encephalitis, myelitis, neuropathy, Lambert-Eaton Syndrome	Not definitely known; probably highly associated with SCLC and other neuroendocrine tumors.
	ANNA3	Combined paraneoplastic cerebellar ataxia with→	limbic or brainstem encephalitis, myelitis, neuropathy	Not definitely known; probably highly associated with SCLC and other neuroendocrine tumors.
	Zic4	In patients with isolated Zic4 abs, mostly paraneoplastic cerebellar ataxia	Associated with various paraneoplastic neurologic syndromes especially if cooccurring with CRMP5 or Hu abs.	> 90%, usually SCLC
	GABA_B_R	Isolated or combined ataxia with→	brainstem encephalitis/ encephalitis with opsoclonus, chorea and seizures	> 50%, often SCLC especially if combined with antibodies against intracellular antigens
	CV2/CRMP5	Combined paraneoplastic cerebellar ataxia with chorea and other clinical features like →	Cognitive decline, neuropathy, optic neuritis, myelitis,	> 90%, SCLC, other neuroendocrine tumors, breast cancer, lymphoma, thymoma.
Chorea and dyskinesias	NMDAR	Coexistence of chorea, dystonia and stereotypies; often characteristic orofacial and limb dyskinesias	Behavioral changes, psychiatric disorders, cognitive impairment, seizures, mutism, dysautonomia	25–50% of woman have ovarian teratomas. In children very rare. In patients > 45 yrs. Other tumors possible, e.g. SCLC, breast cancer, etc
	Neurexin-3a	Orofacial dyskinesias combined with other clinical features like→	Encephalopathy, seizures, altered consciousness, memory deficits, agitation	unkown
	CASPR2	Chorea or hemichorea preceding or combined with behavioral changes	Ataxia, pain, sleep dysfunction, autonomic dysfunction, weight loss, limbic encephalitis; male predominance (85%); age > 65 yrs.	20% (mostly thymoma)
	LGI1	Chorea or hemichorea preceding or combined with cognitive impairment and encephalopathy	Limbic encephalitis, often subacute > 3 months. Bradycardia, hyponatremia	< 5%
	IgLON5	Combined chorea/orofacial dyskinesias with other clinical features like→	Sleep disorder, bulbar dysfunction, gait abnormalities, cognitive decline, eye movement abnormalities	Rare, < 5%
	CV2/CRMP5	Combined chorea with other clinical features like→	Cognitive decline, neuropathy, optic neuritis, myelitis, ataxia	> 90% highly associated with SCLC and thymoma
	Hu	Combined chorea with other clinical features like→	Gastrointestinal pseudoobstruction, sensorineuronal hearing loss	> 95%, Highly associated with SCLC and other neuroendocrine tumors
	D2R	Combined chorea in children with→	basal ganglia encephalitis, “Sydenham’s chorea” or in relapses after HSVE	Unknown, very rare in children
Dystonia	NMDAR	Combined dystonia with chorea and stereotypies and signs of encephalopathy; hemidystonia and craniocervical dystonia are rarely main symptoms in children and young adults	Behavioral changes, psychiatric disorders, cognitive impairment, seizures, mutism, dysautonomia	25–50% of woman have ovarian teratomas. In children very rare. In patients > 45 yrs. Other tumors possible, e.g. SCLC, breast cancer, etc
	Ri	Severe jaw-closing dystonia combined with larnygospasm	Limbic/brainstem encephalitis	> 90%, mostly female patients with breast or ovarian cancer
	IgLON5	Rarely combined dystonia (jaw or/and cervical dystonia) with other clinical features like→	Sleep disorder, bulbar dysfunction, gait abnormalities, cognitive decline, eye movement abnormalities	Rare, < 5%
Myoclonus	CASPR2	Paroxysmal myoclonus triggered by walking or orthostatism, spinal segmental myoclonus, generalized myoclonus> mostly combined with other clinical features ->	Ataxia, pain, sleep dysfunction, autonomic dysfunction, weight loss, limbic encephalitis; male predominance (85%); age > 65 yrs.	∼20% (mostly thymoma)
	LGI1	Usually no myoclonus but facial-brachial dystonic seizures (FBDS); FBDS can be misdiagnosed as myoclonus	Limbic encephalitis, often subacute > 3 months. Bradycardia, hyponatremia	< 5%
	GlyR	Myoclonus typically as part of PERM/ SPSD	Hyperekplexia, opisthotonus, autonomic dysfunction, encephalopathy, eye movement abnormalities, brainstem encephalitis	< 10% thymoma, lymphoma, SCLC, breast cancer
	DPPX	Myoclonus with or without hyperekplexia; mostly combined with other clinical features like→	Limbic encephalitis, brainstem disorders, prolonged diarrhea, weight loss, dysautonomia	< 10%, lymphoma
	IgLON5	Combined myoclonus with other clinical features like→	Sleep disorder, bulbar dysfunction, gait abnormalities, cognitive decline, eye movement abnormalities	Rare, < 5%
	GFAP	Combined myoclonus with other clinical features like→	Meningoencephalomyelitis with headache and subacute encephalopathy	20–40%; diverse neoplasms
Parkinsonism	D2R	Very rare; in children parkinsonism combined with→	Encephalopathy	Unknown, very rare in children
	NMDAR	Combined parkinsonism with other clinical features like→	Behavioral changes, psychiatric disorders, cognitive impairment, seizures, mutism, dysautonomia	25–50% of woman have ovarian teratomas. In children very rare. In patients > 45 yrs. other tumors possible, e.g. SCLC, breast cancer, etc.
	Ma2	Combined paraneoplastic parkinsonism with other clinical features like→ generally subacute and rapid progressive course	Hypothalamic-pituitary dysfunction, weight gain, prominent sleep disorders including excessive daytime sleepiness, rapid eye movement (REM) sleep behavior disorder (RBD), narcolepsy cataplexy, and eye movement abnormalities	> 90% testis tumors
	CV2/CRMP5	Combined paraneoplastic parkinsonism with other clinical features like→ generally subacute and rapid progressive course	Encephalopathy, myelitis, optic neuritis, peripheral neuropathy	> 90%, SCLC, other neuroendocrine tumors, breast cancer, lymphoma, thymoma
	IgLON5	Combined parkinsonism with other clinical features like→ PSP-like picture (vertical gaze palsy)	Sleep disorder, bulbar dysfunction, gait abnormalities, cognitive decline, eye movement abnormalities	Rare, < 5%
	CASPR2	Combined parkinsonism with other clinical features like→	Ataxia, pain, sleep dysfunction, autonomic dysfunction, weight loss, limbic encephalitis; male predominance (85%); age > 65 yrs.	∼20% (mostly thymoma)
	LGI1	Combined parkinsonism with other clinical features like→	Limbic encephalitis, often subacute > 3 months. Bradycardia, hyponatremia	< 5%
	DPPX	Combined parkinsonism with other clinical features like→	Limbic encephalitis, brainstem disorders, prolonged diarrhea, weight loss, dysautonomia	< 10%, lymphoma
Paroxysmal movement disorders	LGI1	Characteristic facial-brachial dystonic seizures (FBDS)	Limbic encephalitis, often subacute > 3 months. Bradycardia, hyponatremia	< 5%
	CASPR2	Paroxysmal episodic ataxia and myoclonus often triggered by orthostatism and walking	Ataxia, pain, sleep dysfunction, autonomic dysfunction, weight loss, limbic encephalitis; male predominance (85%); age > 65 yrs.	∼20% (mostly thymoma)
	NMDAR	Paroxysmal dystonic posturing preceding encephalitis	Behavioral changes, psychiatric disorders, cognitive impairment, seizures, mutism, dysautonomia	25–50% of woman have ovarian teratomas. In children very rare. In patients > 45 yrs. Other tumors possible, e.g. SCLC, breast cancer, etc
	AQP4	Painful tonic spasms	Typically occurring in demyelinating diseases, in particular in NMOSD	< 5%
Stiff person spectrum disorders	GAD	Isolated or combined SPS	Encephalopathy, ataxia, sensory symptoms, pyramidal signs, dysautonomia, epilepsy; often coexistence with autoimmune diseases like type 1 diabetes, vitiligo etc.	< 5%
	GlyR	Isolated or combined SPS, PERM	Oculomotor disturbance, bulbar symptoms, dysautonomia, pyramidal signs, sensory symptoms, encephalopathy; rarely associated with limbic encephalitis	< 10% thymoma, lymphoma, SCLC, breast cancer
	Amphiphysin	Isolated or combined SPS	Ataxia, sensory ganglionopathy and myelopathy	> 90%, SCLC, other neuroendocrine tumors, breast cancer, lymphoma, thymoma
	DPPX	Combined SPS with prominent hyperekplexia, myoclonus, ataxia and further clinical signs like→	Dysautonomia, pyramidal signs, sensory symptoms, cognitive problems. Red flags: Prolonged diarrhoea, weight loss	< 10%, lymphoma
	GABA_A_R	Isolated or combined SPS with →	Epilepsy	unknown but probably rare especially in children
	Ri	Combined SPS as part of →	Brainstem encephalitis	> 90%, mostly female patients with breast or ovarian cancer
Tremor	AMPAR, CASPR2, LGI1, DPPX, GABA_B_R, GlyR, mGluR1, NMDAR	Combined tremor syndrome in the context of→	Limbic encephalitis/encephalitis	AMPAR and GABAR-B > 50% SCLC; mGluR1 lymphoma; for other antibodies see above
	GFAP	Combined tremor syndrome often with ataxia and myoclonus in patient with→	Meningoencephalomyelitis with encephalopathy with epilepsy, cognitive or psychiatric problems, myelopathy, or ataxia	20–40%; diverse neoplasms
	Paranodal antigens (CNTN1, NF155, NF140/186, Caspr1)	Disabling limb tremor in the context of→	CIDP	Rare, < 5%

## Conclusion

In summary, autoimmune movement disorders with neuronal antibodies are an expanding field, with an ever-increasing number of new antibodies and syndromes.

In this review we focused on clinical phenomenology of autoimmune movement disorders and highlighted core phenotypes and clinical red flags, which continue to guide the clinician to suspect an autoimmune movement disorder. Therefore, here we discussed how characteristic phenotypes (like FBDS with LGI1-antibodies, or leg myoclonus affecting stance and gait with CASPR2-antibodies) or associated features (combined syndromes, e.g. chorea with neuropathy in paraneoplastic chorea with Hu- or CRMP5-antibodies) will suggest an autoimmune movement disorder. However, all described entities are neuroimmunological diseases which only rarely present with isolated movement disorders and usually further clinical symptoms are present. Testing for neuronal antibodies to ascertain the diagnosis comes with its own pitfalls. Testing serum and CSF as well as the use of different test systems help avoiding wrong positive and wrong negative test results.

## Data Availability

Not applicable.

## Abbreviations

Ab: Antibodies
AMPAR: α-amino-3-hydroxy-5-methyl-4-isoxazolepropionic acid receptor
ANNA-1: Anti-neuronal nuclear antibody-1
AQP4: Aquaporin 4
CASPR1: Contactin-associated-protein-1
CASPR2: Contactin-associated-protein-2
CBA: Cell-based assays
CBD: Corticobasal degeneration
CIDP: Chronic inflammatory demyelinating polyneuropathy
CMUA: Continuous motor unit activity
CNTN1: Contactin-1
CRMP-5: Collapsin-response-mediated protein-5
CSF: Cerebrospinal fluid
CV2/CRMP5DNER: Delta/notch-like-epidermal-growth-factor-related-receptor
D2R: Dopamine receptor 2
DPPX: Dipeptidyl-peptidase-like protein-6
DYT1: Torsion dystonia-1
ELISA: Enzyme-linked-immune-tests
FBDS: Faciobrachial dystonic seizures
FLAIR: Fluid attenuated inversion recovery
GABA(b): Gamma-aminobutyric acid
GAD65: Glutamate decarboxylase isotype 65
GFAP: Glial-fibrillary-acidic-protein
GlyR: Glycin receptor
GlyRα1: Glycine-receptor-alpha-1
HSVE: Herpes simplex virus encephalitis
LEMS: Lambert-Eaton myasthenic syndrome
LGI1: Leucine-rich-glioma-inactivated-1
mGluR1: Metabotropic glutamate receptor 1
MOG: Myelin-oligodendrocytic glycoprotein
MRI: Magnetic resonance imaging
MSA: Multisystem atrophy
NF155: Neurofascin-155
NMDAR: Anti-N-Methyl-D-Aspartate-receptor
NMOSD: Neuromyelitis optica spectrum disorders
NREM: Non-rapid eye movement
OMS: Opsoclonus-myoclonus syndrome
PANDAS: Paediatric autoimmune neuropsychiatric disorders associated with streptococcal infections
PCD: Paraneoplastic cerebellar degeneration
PD: Parkinson’s disease
PERM: Progressive encephalomyelitis with rigidity and myoclonus
PSP: Progressive supranuclear palsy PTS Painful tonic spasms
RBD: Rapid eye movement sleep behavior disorder
REM: Rapid eye movement
RIA: Radioimmunoassay
SCLC: Small-cell-lung cancer
SLS: Stiff-limb syndrome
SPSD: Stiff-person-spectrum disorders
TBA: Tissue-based assays
TOR1A: Torsin-1a
TRIM9/67: Tripartite motif protein 9/67
VGCC: Voltage-gated-calcium-channels
ZIC4: Zinc finger protein 4
